# Molecular Pathways Involved in Pregnancy-Induced Prevention Against Breast Cancer

**DOI:** 10.3389/fendo.2014.00213

**Published:** 2014-12-10

**Authors:** Maria Barton, Julia Santucci-Pereira, Jose Russo

**Affiliations:** ^1^The Irma H. Russo, MD Breast Cancer Research Laboratory, Fox Chase Cancer Center, Temple University Health System, Philadelphia, PA, USA

**Keywords:** pregnancy, chromatin remodeling, prevention, epigenetics, genomics, differentiation, gene expression, immune response

## Abstract

Pregnancy produces a protective effect against breast cancer in women who had their first full term pregnancy (FTP) in their middle twenties. The later in life the first delivery occurs, the higher the risk of breast cancer development. Also, transiently during the postpartum period, the risk of developing breast cancer increases. This transient increased risk is taken over by a long-lasting protective period. The genomic profile of parous women has shown pregnancy induces a long-lasting “genomic signature” that explains the preventive effect on breast cancer. This signature reveals that chromatin remodeling is the driver of the differentiation process conferred by FTP. The chromatin remodeling process may be the ultimate step mediating the protection of the breast against developing breast cancer in post-menopausal years.

## Introduction

Breast cancer affects women of all ages, races, and nationalities (1–3). The worldwide incidence has increased 30–40% since the 1970s (1, 3–6). In the USA only, it is estimated that there will be around 295,000 new cases of breast cancer in women in 2014 (7). In the same year, 40,000 women in the USA will die of this disease (7). Each year, approximately 13,000 women under the age of 40 are diagnosed with breast cancer. Of these, 1,000 will die of this disease (8).

Epidemiological, clinical, and experimental data have uncovered that one of the risks of developing breast cancer is the reproductive history (9–14). Pregnancy exerts a protective effect in women whose first child was born from their late teens to their middle twenties. This protection is relative to the risk for nulliparous (no offspring) women (9). The postponement of the first delivery increases the risk of developing breast cancer. This risk reaches the same levels observed in nulliparous women when first full term pregnancy (FTP) occurs between 30 and 34 years of age, increasing even further after 35 years (9, 10).

The aggressive form of breast cancer called triple-negative breast cancer is more common in young women under the age of 40. It is not understood why young women are more likely to be diagnosed with this aggressive form of breast cancer (15).

Despite the decrease of risk for breast cancer in lifetime, approximately 30% of breast cancer patients are diagnosed up to 5 years after giving birth (16). This window of susceptibility toward a higher risk of developing breast cancer comes with a greater risk of developing metastasis (16).

This review will address the mechanisms that determine the long-lasting preventive effect of pregnancy against breast cancer, and the transient increase of risk in the years that follow pregnancy.

## Full Term Pregnancy Reduces Breast Cancer Risk

In experiments performed in rats, which is the gold standard animal model for induction of mammary gland differentiation, pregnancy (which takes 21 ± 3 days) has to be completed in order to prevent carcinogen-induced mammary cancer development. In Sprague-Dawley rats, it has been demonstrated that when their first pregnancy was interrupted 12 days after conception and cancer was induced by 7,12-Dimethylbenz(a)anthracene (DMBA) 21 days later, the number and weight of the tumors per animal in pregnancy-interrupted rats and age-matched virgin rats were similar. However, rats that completed their pregnancy had a significantly reduced amount of tumors (17). Completion of the first pregnancy results in significant differentiation of the mammary gland. This differentiation advances even further with milk production and secretion and persists until weaning (17, 18). After weaning, the regression of the lobular structures occurs, and the remaining cells exhibit acquisition of new features such as proliferative rate reduction and increased capacity to repair carcinogen-damaged DNA (17). These new features, which are structural, functional and molecular in nature, persist in the mammary gland, resulting in a significant reduction in cancer incidence in several rat and mice strains (19, 20). Gene expression analysis of the rat mammary gland identified a genomic signature that clearly distinguishes nulliparous (no offspring) from parous rodents. This gene expression profile explains the almost total refractoriness of the parous rat mammary gland to develop neoplasms after carcinogen exposure (19, 21).

Epidemiological studies have demonstrated that a female’s reproductive history is closely linked to breast cancer risk (9, 10, 13). The first FTP is an essential step for determining the fate of the mammary gland in subsequent decades. Pregnancy exerts a protective effect in women whose first child is born before the female reaches her mid-twenties (9, 22). Moreover, multiple FTPs significantly decrease the risk even further, whereas postponement of the first delivery to the female’s mid-thirties increases the risk compared to nulliparous women (9, 10). Pregnancy is a hormonally complex process that only succeeds when there is a perfect synchronization of the levels of estrogen, progesterone and human Chorionic Gonadotropin (hCG), hormones that are essential for the maintenance of pregnancy and breast development in preparation for milk production. Primiparous women younger than 25 years of age that have elevated levels of hCG during the first trimester have a 33% decreased breast cancer incidence in their post-menopausal years (13, 23). On the other hand, high estrogen levels have been associated with increased risk of developing breast cancer in pre and post-menopausal women (13, 23–25). Positive feedback of estrogen secreted by ovarian follicles (26), together with the surge of gonadotropin releasing hormone (GnRH) and luteinizing hormone (LH) trigger ovulation (27). After oocyte fertilization and implantation, estrogen and progesterone are supplemented by chorionic gonadotropin. These three hormones and others contribute to stimulate the mammary gland development by undergoing cell proliferation and differentiation of terminal end buds to organized lobular structures. Final differentiation toward preparation of milk production is achieved by secretion of prolactin that stimulates the production of milk and oxytocin that enhances the secretory activity of the alveolar cells in the mature mammary gland (18, 28). Completion of pregnancy and further breastfeeding induce long-lasting anatomic and molecular changes in the mammary gland (19). These changes result in a significant reduction in breast cancer incidence (17, 29–32).

The above mentioned findings show that the first FTP occurring during the high risk susceptibility window, but before exposure to a carcinogen, prevents mammary cancer initiation. This observation is equivalent to the widely reported protective effect of an early first FTP in women (17, 19, 33). Discriminating whether a first FTP (and lactation) produces protection by inducing complete differentiation of the breast, or, on the other hand, increases the chances of developing breast cancer in pre and post-menopausal years, is still an active area of research and debate. In Section “Mechanisms by Which Pregnancy may Protect Post-menopausal Women from Developing Breast Cancer,” we describe how pregnancy-induced epigenetic modifications that occur during the period of high susceptibility lead to increased protection against breast cancer.

## Mechanisms by Which Pregnancy may Protect Post-menopausal Women from Developing Breast Cancer

In the past, we have addressed the morphological, physiological, and genomic changes that occur during and as a consequence of pregnancy. This hormonally induced differentiation of the breast stamps a mark on breast cancer risk (21, 30, 33–36). The architecture of the breast of women in their reproductive years is composed of three main lobular structures that are classified on the basis of their degree of development into lobules type 1 (Lob 1), lobules type 2 (Lob 2) and lobules type 3 (Lob 3) (17, 37, 38). The breast of nulliparous women is mainly composed of Lob 1, with moderate formation of Lob 2, structures that appear with successive menstrual cycles. Lob 3 structures can only be seen occasionally during early reproductive years. After menopause, the breast regresses and these results in an increase in the number of Lob 1 as a consequence of the decline in Lob 2 and Lob 3 produced with aging (38).

The breast of both post-menopausal nulliparous and parous women contains mainly Lob1 after the regression of the mammary gland subsequent to lactation in the latter group. However, despite its close morphological resemblance, these Lob 1 structures have to be different biologically as parous and nulliparous women differ in their susceptibility to carcinogenesis (35). This concept has been further clarified by showing differences in cell types between these two groups (39).

**Figure 1:**
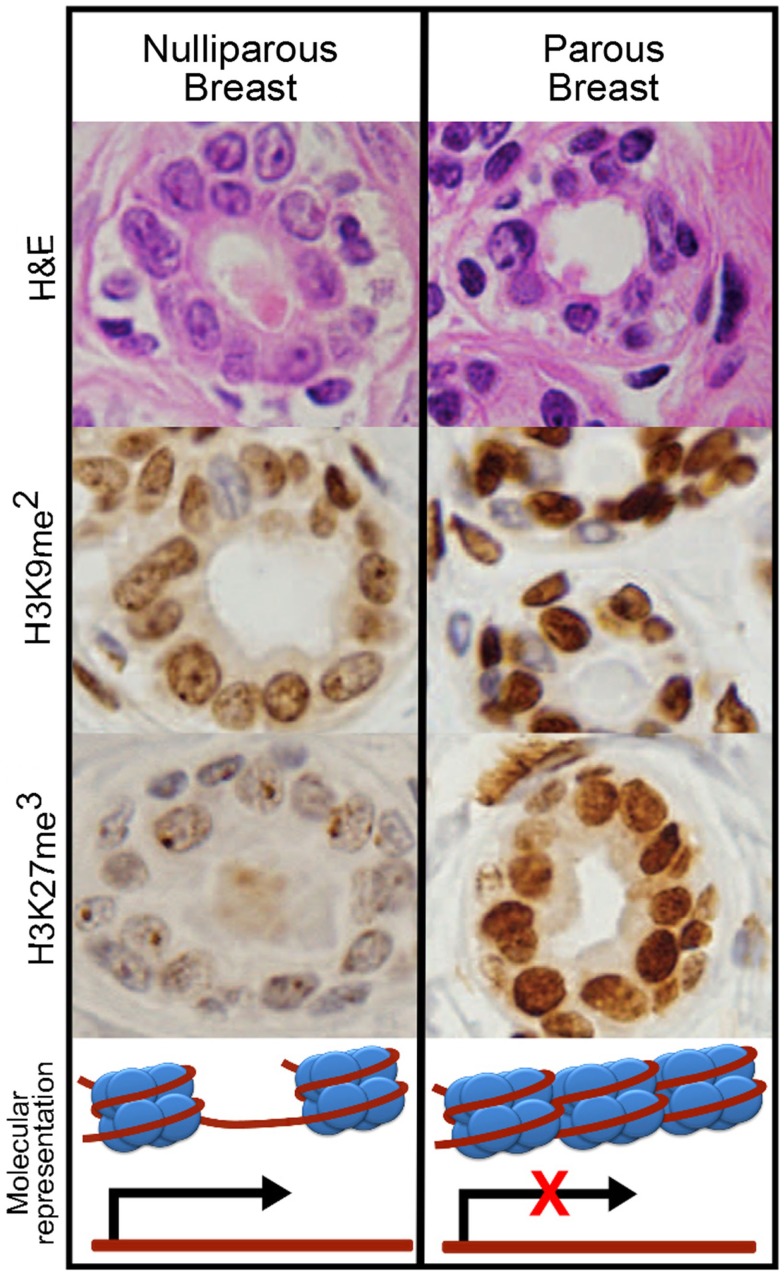
**Epithelial cells of nulliparous and parous post-menopausal breast**. As observed with hematoxylin and eosin staining (H&E), the cells from nulliparous breast (left) contain nuclei with less condensed chromatin (euchromatin) compared to the nuclei of parous women (right). The parous breast contains darker nuclei as a consequence of chromatin compactation (heterochromatin). Immunohistochemistry revealed higher levels of dimethylation of lysine 9 and trimethylation of lysine 27 in histone 3 (H3K9me^2^ and H3K27me^3^) in the parous breast, which have been linked with gene silencing due to chromatin condensation.

The cells from the parous breast contain higher chromatin condensation (heterochromatin), while the breast parenchyma of post-menopausal nulliparous women contains predominantly euchromatin nucleus (EUN) cells (39) (Figure 1), which did not achieve the most differentiated stage due to the absence of a FTP. Therefore, this tissue retains its susceptibility to be transformed. As a consequence, a carcinogenic insult or an inappropriate hormonal stimulus, such as hormone replacement therapy (40), have the potential to transform the EUN cells into a breast cancer stem cell. Thus, a differentiated cell, such as a EUN cell, has the ability to re-acquire self-renewal potential (41, 42). This concept has been further discussed in other experimental models (31, 43–45).

Since the initial finding by Al-Hajj et al. reporting that a small population of CD44^+^CD24^−/low^ breast cancer cells had the ability to form new tumors with as few as a couple hundred cells whereas tens of thousands of cells with alternate phenotypes failed to produce tumors (46), there has been active discussion in the hypothesis of stem cell origin of breast cancer. Normal stem cells self-renew and give rise to phenotypically diverse cells with reduced proliferative potential (47). The tumorigenic CD44^+^CD24^−/low^ cell population is also able to proliferate extensively, and give rise to diverse cell types with reduced proliferative potential (46). By using a xenograft model, Al-Hajj et al. demonstrated that as few as 200 sorted and passaged CD44^+^CD24^−/low^ breast cancer stem cells had the ability to produce tumors that could be serially transplanted in NOD/SCID mice (46). This and other groups have since presented evidence showing that deregulation of self-renewal in stem/progenitor cells may be a key event in mammary tumorigenesis (48–50).

It is generally agreed that the involuted gland in the parous after pregnancy and lactation resembles that of a virgin/nulliparous at the morphological level (51). However, several physiologic, genetic, and epigenetic changes have been noted when comparing parous vs. nulliparous breast epithelium (34, 36, 44). In transplantation studies, Wagner et al. showed that the parity-induced epithelial population acquires self-renewal properties and contributes to the reconstitution of ductal morphogenesis and lobulogenesis at post-weaning (44). This period of lobular regression is accompanied by a reduction in the proliferative rate, greater capabilities of DNA repair and lower DNA affinity for carcinogens (52). It has been proposed that the initial normal progenitor or stem cell 1 is present in the terminal end buds (35). These cells are thought to give origin to the parenchymal tree but when these stem cells 1 are hit by a carcinogen, they become cancer stem cells. With aging and in the absence of previous pregnancy/pregnancies, the parenchyma remains undifferentiated and susceptible to carcinogens. Early pregnancy induces the differentiation of the normal stem cell 1 or progenitor cell into a more differentiated stem cell 2, which is resistant to transformation. This stem cell 2 is still capable of regenerating the gland on subsequent pregnancies in preparation for lactation (35). Gene analysis has identified a “genomic mark” that is sufficient to differentiate nulliparous from parous tissue (34, 35, 44, 53, 54). These differences help to explain the high refractoriness of the parous mammary gland to develop carcinomas (19, 21, 39, 52, 53). This genomic signature in the parity-induced cell population contributes significantly to the biological differences between the mammary glands of parous and nulliparous females, differences, which are long-lasting and thus protect parous women from developing breast cancer.

PLU-1 is expressed in 90% of breast carcinomas and is associated with malignant progression (55, 56). This nuclear protein belongs to the ARID family of proteins, known to play essential regulatory roles in development, differentiation, and chromatin remodeling (57). In the normal mammary gland, *mPlu-1* mRNA is expressed at pregnancy, suggesting a role in proliferation in the developing and differentiating mouse tissue (56). Microarray analysis, after over expression or silencing of PLU-1, has identified specific genes downregulated in mammary epithelial cells such as BRCA1 and genes associated with cell cycle and spindle checkpoints, confirming PLU-1’s function as a transcriptional repressor. Barrett et al. investigated the interactions of PLU-1 with HDACs and their interacting co-repressors. They demonstrated that PLU-1 interacts directly with class I and II HDACs (58). Physiological relevance of this protein in the mammary gland has been reported as it is only expressed in pregnancy and regressing mouse mammary gland but it is silenced during lactation (58). Significantly, high expression of this protein is seen in the great majority of breast cancers (55, 56).

During mammary remodeling related to the menstrual cycle, pregnancy, and lactation, hormones contribute to the development of a mature mammary gland with a definite structure (18, 59–62). Trithorax (TrxG) and Polycomb (PcG) group proteins are required for gland preservation, acting epigenetically by regulating gene expression through DNA methylation, histone modification, and chromatin remodeling (63, 64). Perturbations in these epigenetic regulators are linked to the disruption of epithelial cell identity and mammary gland remodeling, leading to breast cancer initiation (65).

Consistently with higher levels of chromatin condensation observed in the parous groups, the epithelial cells of this group present more dimethylation of histone 3 in lysine 9 (H3K9me^2^) and trimethylation of histone 3 in lysine 27 (H3K27me^3^) (39) (Figure 1). Histone methylation plays a major role in marking transcriptionally active and inactive regions of the genome and associated chromatin, and is crucial in the key events that lead to the development of the mammary gland (66).

Moreover, the parous breast shows up-regulation of chromatin remodeling genes such as Chromodomain helicase DNA-binding protein 2 (CHD2) and Chromobox homolog 3 (CBX3) (36, 39). These proteins are required for controlling recruitment of histones and transcription factors and consequently regulate transcription. CBX3 is involved in heterochromatin-like complexes by recognizing and binding H3 tails methylated at lysine 9. This leads to transcriptional silencing of CBX3 target genes. Two other epigenetic markers related to the PcG that are up-regulated in the parous breast are L(3)mbt-like1 (L3MBTL) and Enhancer of zeste 2 polycomb repressive complex 2 subunit (EZH2) (36, 39). Members of the PcG form multimeric protein complexes that maintain the transcriptional repressive state of genes over successive cell generations. EZH2 is a histone-lysine-*N*-methytransferase, which acts as a gene silencer by adding three methyl groups to lysine 27 of histone 3, a modification that leads to chromatin condensation (67–69).

Recent studies have demonstrated that some non-coding RNA molecules are at the center of nuclear assembly and recruit PcG complexes to the locus of transcription (70). Indeed, up-regulation of long non-coding RNAs (lncRNAs) was observed in the breast of parous women (36, 39). Among the lncRNAs up-regulated in the parous women were XIST (X inactive specific transcript), NEAT1 (Nuclear paraspeckle assembly transcript 1), and MALAT1 (Metastasis-associated lung adenocarcinoma transcript 1) (36, 39). The last two lncRNAs have critical roles in assembly and maintenance of the paraspeckles (71, 72). Further studies evaluated the expression levels of lncRNAs in the breast of healthy post-menopausal women and identified 42 lncRNAs differentially expressed between parous and nulliparous women (73, 74). Of which, 21 were up-regulated and 21 were downregulated in the parous. An additional eight non-coding regions presented statistically significant correlation in expression with their nearby gene, indicating a possible role of the lncRNA as a cis-regulatory element (74). The above evidence places lncRNAs as potential players in the regulation of chromatin transformation that occurs during differentiation.

The spliceosome machinery, stored in the nuclear paraspeckles, plays a critical role in the differentiation process of mouse embryonic stem cells (75). Post-transcriptional modifications of RNA, and recognition by RNA-binding proteins and/or microRNAs are crucial processes in differentiating breast epithelial cells (76). Among the components of the spliceosome that are up-regulated in the post-menopausal parous breast are the heterogeneous nuclear ribonucleoproteins HNRPA3, HNRPA2B1, HNRPD, and HNRPU (36). These HNRPs may be involved in mRNA processing and stability, in addition to other cellular functions such as mammary gland involution, regulation of telomere length maintenance (77), and/or mRNA trafficking (78). Other members of the spliceosome machinery, also up-regulated in the parous breast, are the small nuclear ribonucleoproteins (snRNPs) (36, 39). The snRNPs function as suppressors of tumor cell growth (36, 39) and may have major implications as cancer therapeutic targets. For example, U1 snRNP is an essential component of the splicing complex and has a key role in mRNA processing. Manipulating U1 snRNP’s function could lead to therapeutic purposes in cancer (79). Last, another component of the spliceosome complex that regulates genes involved in the apoptotic process is RBM5 (RNA-binding motif protein 5) (36, 39). RBM5 overexpression causes cell cycle arrest, apoptosis, and inhibition of tumor growth (80). It is also reported to enhance p53-mediated inhibition of cell growth and colony formation (81). Part or all of these mechanisms could also operate in the parous breast.

Cyclin L1 (CCNL1) and L2 (CCNL2) interact with splicing factors localized in the nuclear speckles (82). The epithelial cells in the Lob 1 structures of the parous breast have been reported to present overexpression of CCNL2 protein (39). CCNL1 and CCNL2 are transcriptional regulators which participate not only in the pre-mRNA splicing process (82) but also in the expression of factors that lead to tumor cell growth inhibition and programed cell death, possibly through the Wnt signaling transduction pathway (82, 83). Of interest, the Wnt/β-catenin signaling pathway was found differentially methylated between parous and nulliparous women, indicating a lower production and accumulation of β-catenin in the parous women (84, 85). This decrease in β-catenin may be a leftover effect from mammary involution, which may represent an additional safeguard mechanism occurring in the last steps of mammary gland remodeling (84, 85).

The chromatin modifications observed in the parous breast are accompanied by higher expression of genes related to cell adhesion and differentiation, such as laminins, desmocollin-3, cytokeratin 5, and GATA binding protein 3 (GATA3) (34, 36, 39).

Finally, numerous downstream genes that are regulated by the estrogen receptor α (ER-α) were found to be up-regulated in the parous breast (36). Among these was GATA3, gene that encodes for a protein which belongs to the GATA family of transcriptional regulators. GATA family regulates T lymphocyte differentiation and maturation. Specifically, GATA3 is key to the morphogenesis of the mammary gland, driving the differentiation of progenitor cells (86). It is also a putative tumor suppressor (86). Therefore, the observation that genes involved in the ER regulated pathways are up-regulated in the parous breast suggests that ER-α mediated genes could be under permanent transcriptional modification as a manifestation of a higher degree of cell differentiation.

The regulatory mechanisms highlighted in this section are key to the decrease in susceptibility of the epithelial cell to carcinogenesis. However, more studies need to be conducted to identify the specific pathways involved in this process. Data discussed here emphasizes the relevance of transcriptional and post-transcriptional regulatory mechanisms as critical to the differentiation of the breast. Increasing amount of data is revealing how the combination of genetic and epigenetic modifications is responsible for driving transformation, eventually leading to cancer. Therapeutic strategies that target a combination of genetic mutations together with chromatin modifications, splicing factors, and lncRNA regulation will lead to more effective treatments. In addition, agents which induce changes in the breast cells similar to those induced by pregnancy could potentially be used to protect healthy women considered at high risk of breast cancer (BRCA 1/2 mutation carriers). One example is hCG, which induces cell differentiation in the mammary gland in Sprague-Dawley rats (87).

## Basis of the Dual Effect of Pregnancy in the Premenopausal Woman

The differences in gene expression between parous and nulliparous women were also studied in premenopausal women (88). Gene expression profile of breast tissue from 30 nulliparous and 79 parous premenopausal volunteers between the ages of 30 and 47 years of age, who were free of breast pathology at the moment of biopsy, was analyzed. Because of the known transient increase in breast cancer risk preceding the long-term protective effect of FTP, the authors also examined gene expression differences in parous vs. nulliparous women as a function of time since last FTP. The results show 286 genes differentially expressed (fold-change ≥1.2 and false discovery rate <10%) comparing all parous vs. all nulliparous, and/or, parous women whose last FTP was less than 5 years before biopsy vs. all nulliparous women. Among these, 238 genes were up-regulated, and 48 genes were downregulated in parous compared to nulliparous breast. Of interest is that the up-regulated genes presented three expression patterns: (1) transient: genes up-regulated after FTP but whose expression levels rapidly returned to nulliparous levels. These genes were mainly related to immune response; (2) long-term changing: genes up-regulated following FTP, whose expression levels decreased with increasing time since last FTP but did not return to nulliparous levels. These genes included genes related to immune response and development; (3) long-term constant: genes that remained up-regulated in parous compared to nulliparous breast, independent of time since last FTP. These genes were mainly involved in developmental processes, cell differentiation, and chromatin remodeling. This study shows that a FTP induces long-term expression changes in genes related to the processes of development, cell differentiation, and chromatin remodeling (88) as has also been found in the parous post-menopausal breast (34, 36, 39, 84).

It is not surprising that during the first 5 years after FTP, activation of several genes related to immune response is observed. Growth factors, hormonal signaling and cytokines/chemokines are known to participate in mammary gland differentiation and lactation (89). However, these transiently activated genes may play a role in the short-term increase of breast cancer risk following FTP (88). Some of these genes showed large differences in expression among the parous women, and that could be one of the explanations why some women develop breast cancer soon after their FTP. Rotunno et al. studied the gene expression between parous and nulliparous, including premenopausal women (mean age = 37), and observed a significant amount of genes associated to immunity, inflammation, and wound responses (90). The inflammatory microenvironment as well as the wound response genes could contribute to the development of breast cancer in certain women (90, 91).

## Conclusion

Pregnancy exerts a protective effect in women who had an early FTP. However, approximately 30% of breast cancer patients are diagnosed up to 5 years after giving birth (16).

The genomic profile of nulliparous and parous women in the premenopausal and post-menopausal period have shown that some groups of genes are only activated during the first years after FTP (88), while others are part of a long-lasting signature (34, 36, 39, 84). Genes, which are only activated during the first 5 years after pregnancy (88), may contribute to the increased risk experimented by some women after pregnancy. On the contrary, the long-lasting signature induced by the FTP observed in the pre and post-menopausal women explains pregnancy’s preventive effect. Evidence point toward chromatin remodeling being the major molecular mechanism that explains pregnancy’s preventive effect (39, 84) (Figure 1). A better understanding of the molecular effects of parity on the breast may help the development of novel strategies for preventing breast cancer.

## Conflict of Interest Statement

The authors declare that the research was conducted in the absence of any commercial or financial relationships that could be construed as a potential conflict of interest.
